# Envisioning the next human genome reference

**DOI:** 10.1242/dmm.049426

**Published:** 2021-12-22

**Authors:** Monkol Lek, Elaine R. Mardis

**Affiliations:** 1 The Anlyan Center, Yale School of Medicine, New Haven, CT 06520, USA; 2 Institute for Genomic Medicine, Nationwide Children's Hospital, Columbus, OH 43215, USA

## Abstract

**Summary:** We provide an Editorial perspective on approaches to improve ethnic representation in the human genome reference sequence, enabling its widespread use in genomic studies and precision medicine to benefit all peoples.

This year marks the 20th anniversary of the announced completion of the draft human genome sequence. The reference genome was a transformative accomplishment for human biological and medical research and is often referred to as biology's moonshot. Over the past 20 years, the availability of this reference, and its refinement, has had the predicted transformative impact on our understanding of human genetic diseases, and, in many ways, has revolutionized the practice of medicine and medical diagnosis. These advances are mainly due to the emergence and refinement of rapid sequencing technology, which has facilitated our ability to generate genomic data, and to corresponding advances in computational analysis of these data, which have solidified the significant role of genomic alterations in disease etiology. Along these lines, large international projects have enriched our understanding of human genomic diversity in the context of cancer (Campbell et al., 2020), psychiatric genetics (Bipolar Disorder and Schizophrenia Working Group of the Psychiatric Genomics Consortium, 2018; Cleynen et al., 2021), autism (Satterstrom et al., 2020; Trost et al., 2020) and many other diseases.

Such studies have mainly catalogued individual and ancestry-based variation in human genomes, albeit to a limited extent. The inherent limitation in scope has been reflective of a predominant focus on populations of European ancestry in the earliest studies, such as the 1000 Genomes Project. More recent attempts to address these disparities have illuminated the challenges in recruiting diverse populations that either have a justifiable lack of trust in medical research or have cultural complexities regarding consent to participate. By improving diversity and inclusion in these studies, there would be increased hope that genomic studies will more broadly benefit populations. In order for the human genome and genomics to have a more significant and equitable impact in the future, accordingly, there is much more to be done. Medical and scientific communities need to consult with and listen to diverse populations and cultures to understand their concerns and needs, and, importantly, to make corresponding changes in our research practices that ensures accountability to these groups.

**Figure:**
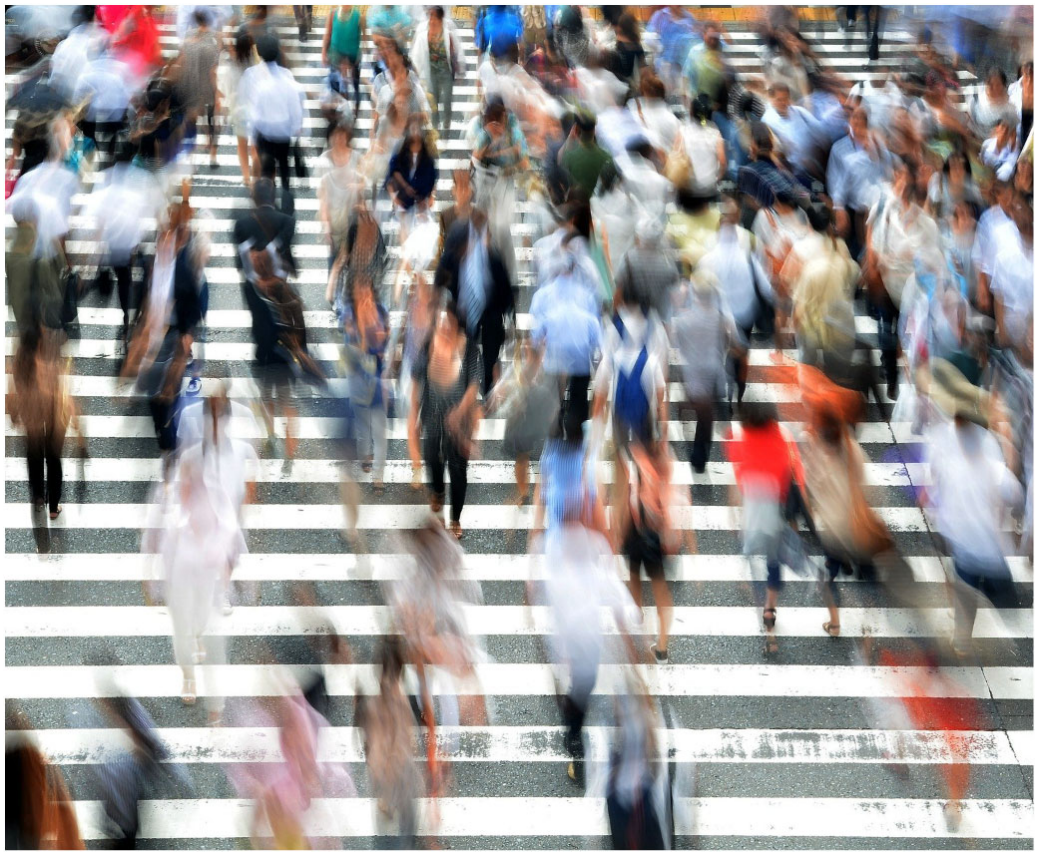
Image reproduced under the terms of the Pixabay License.

Ongoing large-scale population studies that link with individual clinical information are beginning to shape newer capabilities in predicting complex genetic disease susceptibility, primarily focused on developing and testing polygenic risk scores (Shen et al., 2020; Vujkovic et al., 2020; Riveros-Mckay et al., 2021; Weale et al., 2021). These efforts have the potential to radically change the practice of medicine, from a reactive to a proactive model of care delivery, based on an individual's likelihood to experience predicted health challenges during their life course. As such, not only is current genomics-based diagnosis being imperiled by a lack of understanding of genomic variation based on ancestry (Sirugo et al., 2019), but future health care aspects also will be compromised in an ancestry-specific way, further compounding the impact of systemic racism that continues to make ethnic minorities more vulnerable in this setting (Yudell et al., 2020). In aggregate, this means that health disparities in many underserved populations will continue. In addition to these disparities in genomic representation, we also still lack a fundamental understanding across all ancestries, of what genotypes define as ‘healthy’ relative to our significant and growing understanding of ‘diseased’.

There is renewed hope as newer projects strive to improve inclusivity, and here we highlight several examples arising in the United States. The National Institutes of Health (NIH)’s All of Us Research Program has set laudable goals for inclusion of diverse ethnic groups based on recent metrics, wherein over 50% of the currently enrolled 394,000 participants are ethnic minorities. This is a promising beginning, and, with a planned recruitment of 1 million participants, it will be interesting to see the final percentages toward the stated goal. More recently, the NIH posted a request for applications in support of research leading to the creation of best practices for the study of population identifiers. Local projects that focus on specific populations are also emerging, with a variety of funding mechanisms. For example, a study funded by the New York Genome Center plans to enroll minority participants from the broad diversity found throughout the New York City metropolitan area, with an aim to sequence whole genomes and collect health-related information, as discussed by Harold Varmus, one of the study's leaders (Varmus, 2019). In Columbus, Ohio, the Institute for Genomic Medicine has opened an Institutional Review Board-approved study to consent, produce and database whole-genome sequences from unrelated individuals of Somali descent, to better inform our genomics-based diagnostic efforts for Somali children. Similarly, at Yale University, the Generations Project aims to increase diversity by recruiting in the Connecticut area, which is closely aligned with diversity in US consensus metrics. The exome sequencing and genotyping data from this project will be linked to electronic health records, allowing an opportunity to study and advance genomic health in under-represented minorities. Olufunmilayo Olopade, at the University of Chicago, has also discussed her work investigating genetic risk factors for breast cancer in Black women in studies based in Chicago and West Africa that aim to improve early detection, prevention and treatment in these populations (Olopade, 2021). Similar efforts outside the United States include the Human Heredity and Health in Africa Initiative (H3Africa), GenomeAsia 100K, ChileGenomico and the oriGen Project based in Mexico, among others.

Technology continues to impact our human genome reference, predominantly using long-read single-molecule sequencing technologies to generate data, and algorithms capable of assembling these reads into long stretches of human chromosomes, permitting a more complete understanding of structural variation and unique content. Recently, an ‘end-to-end’ assembly of a complete hydatidiform mole cell line was reported, providing contiguity across centromeric and other complex repeats in the human genome, as described in a preprint (Nurk et al., 2021). However, the ancestry of this sample is European. Importantly, the Pangenome Project will aim to produce high-quality long-read sequencing for 300 individuals originally profiled in the 1000 Genomes Project. These comprehensive reference genomes will also include sequences of highly repetitive regions, including centromeres, segmental duplications and ribosomal DNA (rDNA) arrays on telomeres. In addition, the population diversity provided by these genomes will give researchers the option to choose a reference that is more closely related to any given sequenced individual, resulting in improved variant discovery.

In addition to the influence of genomic technology, we have branched out from sole focus on DNA sequence to cataloguing RNA expression, isoforms and other types of characterization by applying next-generation and single-molecule sequencing, which, when integrated with DNA information, can provide significant insights into the sequences actively being expressed in tissues, as well as those being silenced by chromatin conformation or methylation. Such studies reveal that there is much more to be learned, and emphasize the importance of cataloguing normal tissue gene expression, which is available at GTEx, the Allen Brain Atlas and other internet resources. Exquisite new knowledge of gene expression profiles at the single-cell level from normal and diseased human tissue is emerging from the Human Cell Atlas projects, revealing the intricacies of human biology at high resolution (Ponting, 2019; Lindeboom et al., 2021).

Yet, the concern about inclusion persists, even for these newest technological avenues that may indeed reveal important, ancestry-relevant differences with respect to disease susceptibility, physiologic specificity, pharmacogenomics and other pertinent areas (Okada et al., 2018). Without broadening the scope of diversity, we are concerned that individuals and populations will be left behind in many aspects of genomic medicine, effectively broadening disparities. Considering worldwide disparities that exist, such as poor access to health care, even in countries with high income and/or universal health care, the question remains about how to effectively foster inclusion and ensure that under-represented populations will benefit from expanded genomic research. Several strategies for increasing diversity and inclusion have been published (Cooke Bailey et al., 2020; Essien and Ufomata, 2021; Rotimi and Adeyemo, 2021; Nature Editorial, 2021). Here, we would like to highlight the following approaches. First, diversity in research subjects and samples starts with a diverse workforce at all levels, including leadership of major consortium efforts. To enable the creation of a diverse workforce, researchers need to engage with these communities to encourage and support them in pursuing such career paths and overcoming institutional barriers to achieve these goals. Researchers from under-represented groups that truly understand the communities being studied should have the opportunity to participate at all levels of a project, including leading the project and consenting participants (Bonham and Green, 2021). This facilitates surmounting socio-economic and cultural barriers that make it difficult to recruit under-represented minorities and engages the study team to meet diversity recruitment goals, while ensuring that the interpretation and outcomes of research are broadly beneficial. Second, there needs to be increased funding of institutions with diverse staff and students, such as Historically Black Colleges and Universities or community colleges with 2-year associate programs, as well as internship programs that bring minority students from inner-city high schools into genomics laboratories to learn about genomics research and its applicability to human health. Such programs ensure that leadership opportunities and training in genomics will directly benefit the communities that we intend to recruit. Equally important in this regard is education directed at researchers and medical providers that illuminates ongoing issues concerning racism, diversity and inclusion in science and health care. Third, long-term funding must be dedicated to build infrastructure and collaborative networks to enable the facile recruitment of diverse cohorts. H3Africa is a good model that could be replicated in other under-represented regions across the world, including diverse populations in large inner-city settings, emphasizing needed focus on consultation within these communities to understand their wants, needs and concerns regarding genetics and genomics. Lastly, funding agencies need to switch mindsets from having diversity as an optional goal to being a measurable milestone that must be met. The Human Cell Atlas in their recent funding round for the Pediatric Cell Atlas provides one example of explicit ancestry recruitment goals. Longer term, we must take responsibility to put in place mechanisms that both ensure accessibility to data and quantify the benefit of these studies to all populations.

In reflecting on the 20 years since the published draft human genome, it is time to recognize that the combination of technologies, computational algorithms, and the diversity and inclusion of participants gives us the opportunity, this time around, to design cohort studies to benefit ALL of us. Certainly, there is a responsibility for journals, such as DMM, to address the issue of diversity and inclusion, by encouraging the publication of research that advances our understanding of diseases over-represented in individuals of diverse ancestries, and also encouraging reviewers to be conscious that access to technology is not equivalent in all countries, when requesting revisions for publication. DMM's policies include aims to engage diverse and inclusive groups of authors, reviewers, Editors, Editorial Board members, readers and the communities being studied, and the journal is a signatory of the Royal Society of Chemistry's initiative ‘Joint commitment for action on inclusion and diversity in publishing’. Journals can also seek out and publish pieces that address these important (and sometimes difficult) conversations, and openly discuss the ongoing challenges as well as approaches employed by others, as we strive to identify solutions that benefit everyone.
